# Exploring the effect of soybean fermentation broth (S-FB) on gut microbes of lipopolysaccharide (LPS)-infected loach (*Misgurnus anguillicaudatus*) using 16S rRNA sequencing

**DOI:** 10.3389/fmicb.2025.1551409

**Published:** 2025-03-18

**Authors:** Shenghui Chu, Ruike Fan, Lishang Dai, Min Liu

**Affiliations:** ^1^Key Laboratory of Xinjiang Phytomedicine Resource and Utilization, School of Pharmacy, Ministry of Education, Shihezi University, Shihezi, China; ^2^School of Pharmaceutical Sciences, Wenzhou Medical University, Wenzhou, China; ^3^School of Traditional Chinese Medicine, Wenzhou Medical University, Wenzhou, China

**Keywords:** soybean fermentation liquid, loach, 16S rDNA sequencing, intestinal microbiota, lipopolysaccharide

## Abstract

The fermentation products of soybean are rich in beneficial bacteria, which play Shenghui Chu a significant role in maintaining the balance of intestinal microbiota and improving intestinal health. To investigate the immunomodulatory effects of soybean fermentation broth (S-FB) on loach *(Misgurnus anguillicaudatus)* with lipopolysaccharide (LPS)-induced enteritis, 16S rDNA high-throughput sequencing technology was employed to analyze the composition and structure of intestinal microbiota in two groups: the LPS-treated group (fed with soybean broth) and the control group (normal feeding conditions). The results revealed that the relative abundance of beneficial bacteria, such as Lactobacillus and Muribaculaceae, significantly increased in the treatment group, while the relative abundance of harmful bacteria, including Aeromonas and Shewanella, decreased. These findings suggest that soybean fermentation broth can repair intestinal damage and maintain intestinal health by enhancing the abundance of beneficial bacteria and reducing the pathogenic effects of harmful bacteria on the host. Functional prediction studies of microbial communities also showed that treatment groups primarily affected metabolic and genetic information processing. The research results analyzed the changes in the structure and distribution of intestinal microflora in different groups of loach, providing new insights into the possible role of soybean fermentation liquid in intestinal inflammation.

## 1 Introduction

The loach (*Misgurnus anguillicaudatus*), which specifically belongs to the Cobitidae family, is an essential endemic freshwater species and is widely distributed in China, Japan, Korea, and other Southeast Asian countries (Huang et al., 2022; Zhong et al., 2019). Its edible part of the fish accounted for about 80%, higher than the general freshwater fish, delicate meat, delicious taste, rich in nutrients, the water ginseng reputation (Wang et al., 2018; Xu et al., 2016; Zhu and Wu, 2018; Zhu et al., 2016). With the continuous improvement of people’s living standards, loach market demand gradually increased. Loach market prospects will be broader. The loach not only can breathe with gills and skin but also has a characteristic lung respiratory function, so the loach tolerance of low dissolved oxygen is much higher than the general fish, and feeding conditions are lower than the classical fish (Huang et al., 2016; Sun et al., 2021; Sun et al., 2023). Notwithstanding, the growth rate of loach was relatively slow as well as the survival rate of seedlings was low. Owing to that loach lives in the environment of humus underwater, and is easily affected by the temperature. The common diseases of loach include enteritis, skin rot, water mold, and red dot disease, which seriously affect the economic benefits of the farmers (Wang et al., 2020a). Furthermore, the treatment of loach infections with antibiotics often results in the emergence of antibiotic-resistant bacterial strains, the accumulation of residual antibiotics, and ineffective treatment (Zhang et al., 2020). There exist few reports about how to avoid enteritis in the process of raising loach, in the present circumstances, more cost-effective prevention and control methods are urgently needed.

The intestinal flora is remarkably abundant in the digestive tract, including bacteria, fungi, prokaryotes, archaea, and viruses. Dietary fiber, protein, and polypeptides are digested mainly through fermentation and anaerobic degradation and provide energy through metabolism to maintain intestinal health (Acheson and Luccioli, 2004; Dahan et al., 2016; Yang et al., 2022). Current research shows that some metabolic disease symbols include obesity, type 2 diabetes, cardiovascular disease, liver disease, and kidney disease, all of which may be caused by an imbalance in the gut flora or due to an imbalance in the gut flora (Blandino et al., 2016; de Cuevillas et al., 2022; Hsu et al., 2021; Wang et al., 2020b; Witkowski et al., 2020). A healthy gut flora largely determines the overall health of the host. Probiotics contained in the fermentation were clearly expressed as probiotics defined as space “Live microorganisms that, when given in sufficient quantities, are beneficial to the health of the host” that interact with gut symbionts or pathogens to inhibit pathogenic bacteria; To maintain the stability of intestinal flora (Mallon et al., 2015), probiotics can also participate in nutritional metabolism (Derosa et al., 2021), the immune system (Lau et al., 2021), regulation of endocrine and even neuromental health (Lau et al., 2021; Sorboni et al., 2022). As a direct consequence, we put forward a hypothesis: We can upgrade the intestinal flora, by using the benefit of fermentation feed, next to raising better health loaches.

Soybean is a good source of fat and protein for human beings, rich in cellulose and physiologically active substances, and is one of the most determinative grains in China. It is accustomed to generating various bean products, extracting bean oil, brewing soy sauce, and extracting protein (Kim et al., 2015; Kim et al., 2021). Soybean contains many active substances that exist in the plant cells of soybean. Its outer layer is a cell wall composed of cellulose. Mechanical methods can help destroy plant cells, but this is not efficient for extracting the active substances, this problem can be effectively solved by the biochemical action of microorganisms. Microbial activities were essential to increase the bean’s digestibility, nutritional value, and sensory quality, additionally to lower its antinutritive factors (Lee et al., 2019). In addition, in most industrial soybean processing practices, fermentation is a simple and inexpensive technique that can enormously improve the nutrition, texture, and flavor of soybeans, among most of the industrial procedures for soybean processing, fermentation is a simple and inexpensive technique that can substantially improve the nutrition, texture, and flavor of soybean, thus becoming a popular process throughout the world (Liu et al., 2022). Li et al. (2020) found that replacing fermented soy products with traditional fish food enhanced the concentration of probiotic Lactobacillus and anti-inflammatory bacteria Faecalibaculum in flounfish, and inhibited the amount of pathogenic bacteria Vibrio, thus producing an anti-inflammatory effect. Wang P. et al. (2024) found that Monascus purpureus M-32 fermented soybean meal (MFSM) enhanced the resistance of Pacific white shrimp to Vibrio parahaemolyticus infection and enhanced the immune response. The benefit of soybean fermentation liquid as loach feed can not only regulate the micro-balance of nature in aquatic animals, promote the healthy growth of animals, improve production performance, reduce costs, and increase efficiency, but also regulate and protect the aquatic ecological environment. Our country’s aquaculture area is humongous, and the application of fermentative feed raw material conforms to the current healthy environmental protection fishery development request and has a broad market application prospect.

Lipopolysaccharide, a pro-inflammatory compound from the outer walls of Gram-negative bacteria, promotes intestinal inflammation. A connection between low-grade inflammation, sustained by lipopolysaccharides (LPS), and the development of metabolic disorders is well established (Di Lorenzo et al., 2019; Yoshida et al., 2018). In this study, the intestinal flora of the loach was out of balance after LPS treatment, and then the loach was fed with soybean fermentation liquid. The high-throughput sequencing of 16S rDNA was acquainted with to investigate the effect of soybean fermentation liquid on the composition and structure of intestinal microflora in loach. This study will provide a theoretical basis for the advancement of fermented soybean feed, and support a new idea for how to feed more healthy loach. It can be responsible, in addition to this, for fresh insights into the treatment of intestinal diseases from the perspective of fermentation.

## 2 Materials and methods

### 2.1 Preparation and treatment of S-FB

First, the soybean was soaked overnight, and deionized water was added according to the mass ratio of 1:7, and blended to make soybean homogenate. Next, 10 g of homogenate was added to 39.5 g water and autoclaved at 121°C for 20 min to obtain soybean medium. After the medium was cooled, a 0.5 ml OD600 = 0.8 lactobacillus suspension was inoculated and incubated at 37°C for 72 h. The fermented soybean medium was filtered with 0.22 μm microporous filter membrane to remove the bacteria and soybean residue to obtain soybean fermentation solution (S-FB).

### 2.2 Experimental animal and LPS treatment

The experimental loaches were brought trade to the loach breeding base in Wenzhou. A total of 50 loaches with good vitality and unflawed body surfaces were selected, with an age of 6 months, uniform size, body length of 9.00 ± 0.50 cm, and body weight of 8.00 ± 0.20 g. Before the formal start of the experiment, the loaches were contained in a water basin for 7 days for daily feeding. The container length, width, and height were 37.0 cm, 31.5 cm, and 13.5 cm, respectively. The loaches catered according to their weight during the temporary feeding period. The loaches branched into two groups in disorder: the control group and the treatment group, with 25 loaches in each group. Each loach in both the control and treatment groups was intraperitoneally injected with 100 μL of LPS solution [60 mg/100 μL, dissolved in phosphate-buffered saline (PBS)] (Liu et al., 2016). The LPS used in this study was purchased from Beijing Solarbio Science and Technology Co., Ltd. The PBS solution preparation method is as follows: 8.00 g NaCl, 0.20 g KCl, 1.44 g Na_2_HPO_4_, and 0.24 g KH_2_PO_4_ and dissolved in 800 mL of distilled water. Adjust the HCl solution to pH 7.4, and supplementary distilled water to make a constant volume of 1 liter from then on. After injection, the loaches were fed in clean water and soybean fermentation liquid (fermented by lactic acid bacteria), respectively. In the meanwhile, the feed was added. After 24 h, the surface of the loaches was wiped with 75% alcohol, then the loaches’ midgut was taken out and quickly frozen in dry ice under sterile conditions. Consequently, the samples were preserved at −*80*°C until analysis.

### 2.3 DNA extraction, library preparation and sequencing

Microbial DNA was extracted using the HiPure fecal DNA kit (Magen, Guangzhou, China) according to the manufacturer’s protocol. The resulting DNA was then assessed for quality using a NanoDrop-2000 differential photometer (Thermo Fisher Scientific, United States) and 1% agarose gel electrophoresis. The V3–V4 variable region of the 16S rRNA gene was amplified and sequenced using barcode forward primer 341F (5′-CCTACGGGNGGCWGCAG) and reverse primer 806R (5′ -GGACTACHVGGGTATCTAAT), and during the primer amplification, the samples were barcoded. It was then purified with AMPure XP beads, quantified by QuantiFluorTM fluorometer (Promega, United States), and sequenced by Illumina PE250 platform (Illumina, SanDiego, California) to obtain Raw reads.

The raw 16S rRNA gene sequencing data were processed using an open-source bioinformatics pipeline. Quality control (QC) analysis was performed on the raw data, and low-quality reads were filtered out using FASTP software. Merged by fast length adjustment of short reads (FLASH). Operational Taxonomic Units (OTUs) were clustered at a 97% similarity threshold using the UPARSE algorithm implemented in USEARCH software. The chimeric sequences were identified and removed using the UCHIME algorithm during the clustering process. This pipeline yielded effective tags and enabled the statistical analysis of OTU abundance.

### 2.4 Bioinformatics analysis

#### 2.4.1 Species composition analysis

In the process of constructing OTUs/ASVs, selected representative sequences (Tagsequence/ASV consistent sequence with the highest abundance in OTUs and used the RDP Classifier’s Naïve Bayesian assignment algorithm (Wang et al., 2007) to annotate the species with the database (set the confidence threshold to be 0.8–1). According to the species annotation information of OTUs/ASVs, the number of Tags sequences on each taxonomic level (phylum, family, genus, and species) of each sample was counted, and a histogram of the taxonomic composition at different taxonomic levels among each group was drawn.

#### 2.4.2 Species indicator analysis

Use the R language labdsv package (Tareau et al., 2020) to calculate the indicator values of each species in the comparison group in each group. And use cross-validation for statistical testing to obtain a *P*-value. Displayed as a bubble chart, it is possible to visually find the biomarker of each group by bubble size (Chen and Boutros, 2011; Conway et al., 2017).

#### 2.4.3 Alpha diversity analysis

Rarefaction curve analysis: using the relative ratio of known OTUs in the measured sequence, calculate the expected value of the alpha diversity index when extracting n (n is less than the total number of measured tags) tags, and then draw a curve based on a set of *n* values (typically a set of equal difference sequences smaller than the total number of sequences) and their corresponding expected value of the alpha diversity index. When the curve flattens or reaches a plateau, it can be supposed that the sequencing depth has covered all species in the sample.

Rank abundance curve: The method for drawing a Rank abundance curve is to sequence the OTUs in the sample from the largest to the smallest relative abundance (or the number of sequences included) to obtain the corresponding sequence number, and then use the sequence number of the OTUs as the abscissa and the relative abundance in the OTUs (or the relative percentage content of the sequence number in the OTUs of this grade) as the ordinate. Connect these points with a broken line (McMurdie and Holmes, 2013).

#### 2.4.4 PICRUSt and Tax4Fun community function prediction

PICRUSt2 community function prediction: Combine with IMG (Integrated Microbial Genomes) database to collate high-quality bacterial and archaeal genomes, and construct phylogenetic trees based on sequences for functional prediction (Langille et al., 2013). Tax4Fun community function prediction: First, associate the 16S rRNA sequence of prokaryotes with existing genomes in the KEGG database with the 16S rRNA sequence in the SILVA database, and then interrupt the genome of prokaryotes with existing genomes in the KEGG database, using UProC to perform statistics on the KO sequence of all genomes; Finally, use the copy number of 16S to correct the number of species, and implement KEGG prediction and KO abundance statistics in the end (Aßhauer et al., 2015).

#### 2.4.5 Bugbase phenotype classification prediction

On the basis of the green gene database, we use Bugbase to predict the phenotype of the community. By integrating genetic information from IMG, KEGG, and PATRIC databases, they are classified into seven main types: Gram positive, Gram negative, biofilm formation, pathogenicity, mobile elements, and oxygen utilizing, including aerobic: Aerobic, Anaerobic, Facultative anaerobic and oxidative stress tolerance.

### 2.5 Ethical statements

All experiments were conducted in strict compliance with relevant national laws and regulations, as well as ethical guidelines for animal research. Additionally, the animal experiments were reviewed, approved, and supervised by the Experimental Animal Ethics Committee of Wenzhou Medical University to ensure ethical standards and the welfare of the animals were maintained throughout the study.

## 3 Results

### 3.1 Quality control and overview of sequencing data

**FIGURE 1:**
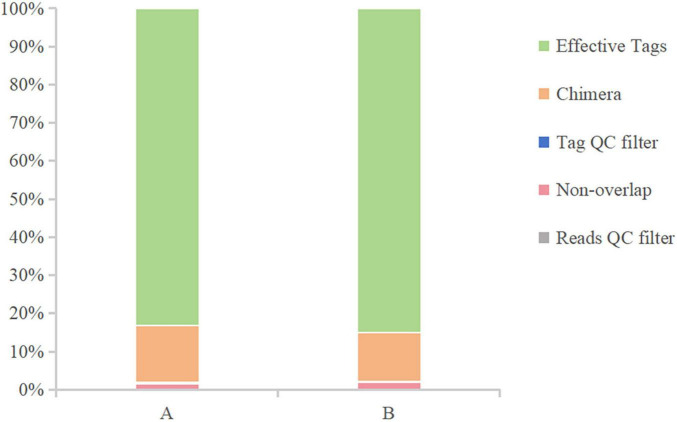
Data preprocessing distribution chart. The X-axis represents the group, and the Y-axis represents the proportion.

**FIGURE 2:**
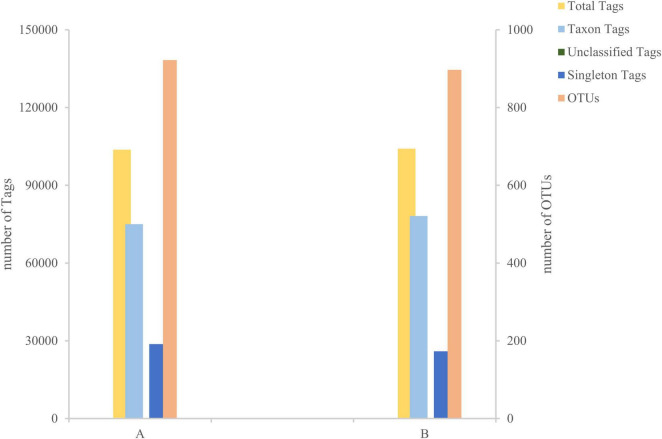
Tags and Operational Taxonomic Units (OTUs) quantity statistics chart. The X-axis represents the group, and the Y-axis represents the number.

After sequencing the midgut samples of loach from groups A and B, 124,819 raw reads were obtained from Group A, and 122,512 raw reads were obtained from Group B, then we perform statistical screening on each sample to obtain pre-processing results (Figure 1). After the original data was pretreated by removing the low-quality fragments, splicing, and chimerism, the effective tags obtained from the control Group A and fermentation liquid treatment Group B were 103,767 and 104,118, respectively, and the effective ratios of the two groups were more extraordinary than 83%. The depth of sequencing can reflect the species of bacteria in the samples, so we believe that the sequencing results are reliable. Then, based on the obtained OTU abundance information and species annotation information, the overall characteristics of OTUs in the two groups of samples were statistically summarized (Figure 2). It was found that the effective tags of groups A and B were not significantly different, but the number of OTUs obtained through layer by layer screening was slightly higher in group A: 922 OTUs were obtained in group A, and 897 OTUs were obtained in group B (Table 1), indicates that the current sequencing quantity can be in preparation for subsequent analysis.

**TABLE 1:** Summary of the sequence analyses.

Sample	Raw reads	Clean reads	Raw tags	Clean tags	Effective tags	Effective ratio (%)	OTU
A	124,819	124,687	122,848	122,514	103,767	83.13	922
B	122,512	122,353	120,262	119,933	104,118	84.99	897

Raw reads: the number of original reads obtained without filtering low-quality sequences. Clean reads: the number of reads after removing low-quality sequences. Raw tags: the number of original tags obtained from the clean reads after overlap assembly. Clean tags: high-quality tag data obtained from raw tags spliced through quality and length filtering processing. Effective tags: the number of high-quality tags after removal of chimeras. The subsequent analysis is based on effective tags. Effective ratio (%): effective tags as a percentage of raw reads. OTU, Operational Taxonomic Units.

### 3.2 Analysis of intestinal microflora in loach

**FIGURE 3:**
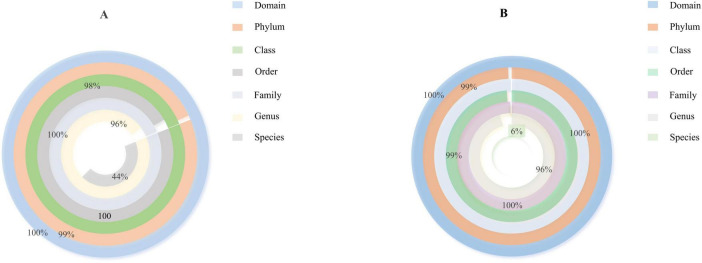
Species annotation tags scale donut chart, according to the species annotation information of Operational Taxonomic Units (OTUs)/ASVs, count the number of Tag sequences for each sample at each taxonomic level (phylum, phylum, family, genus, and species). **(A)** Proportion of species classification in Group A, **(B)** Proportion of species classification in Group B.

**FIGURE 4:**
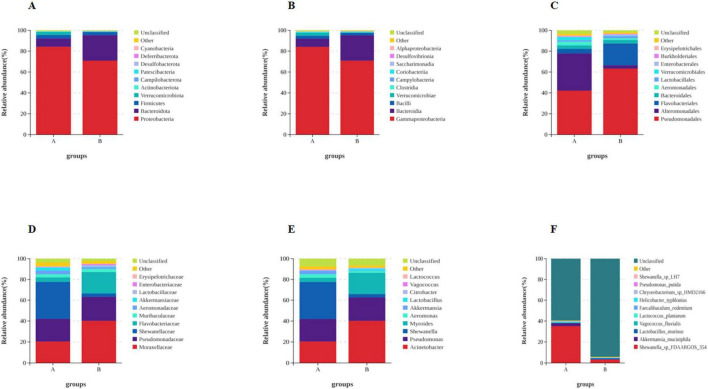
Effect of dietary Soybean liquid supplementation on the relative abundance of gut microbiota in different taxa levels. **(A)** phylum, **(B)** class, **(C)** order, **(D)** family, **(E)** genus, **(F)** species.

**FIGURE 5:**
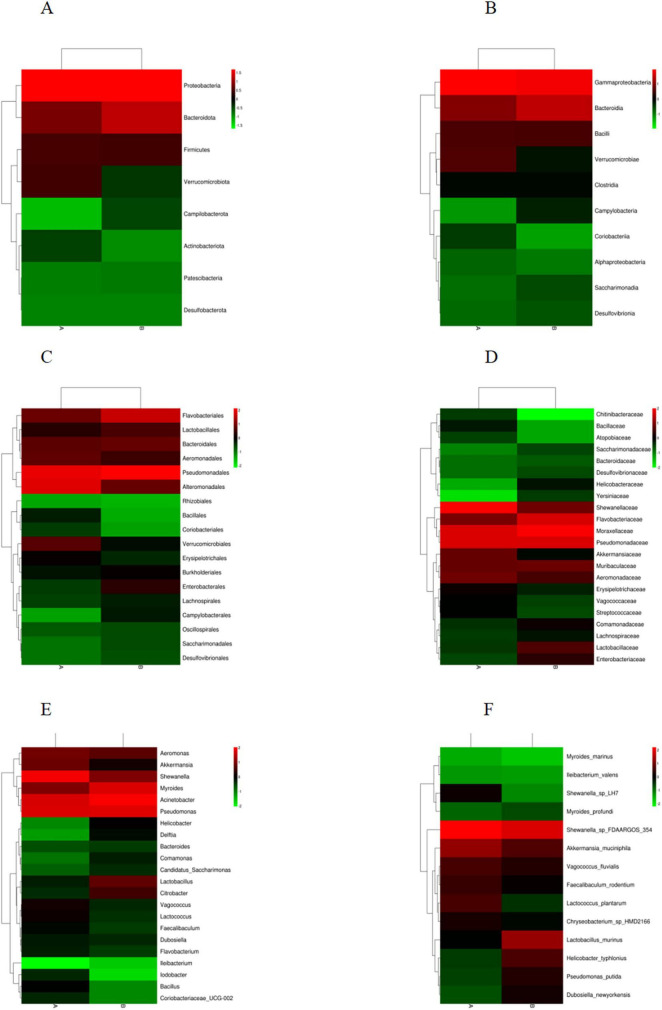
Heatmap of species classification at the level below the boundary. **(A)** Phylum, **(B)** Class, **(C)** Order, **(D)** Family, **(E)** Genus, **(F)** Species.

**FIGURE 6:**
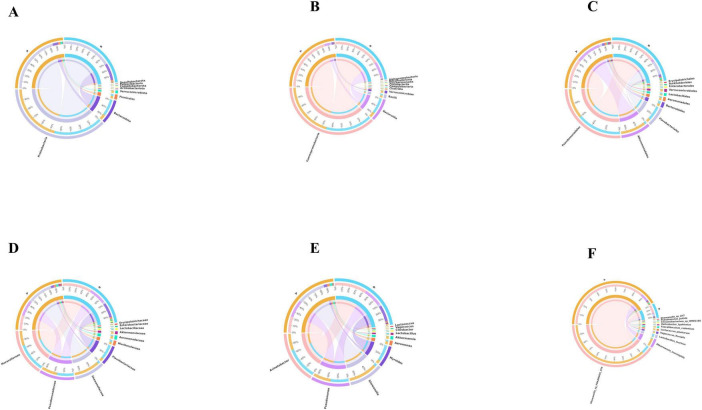
Species distribution circos map. For each classification level, display the top 10 species in abundance among all samples/groups using a circos chart. **(A)** Phylum, **(B)** Class, **(C)** Order, **(D)** Family, **(E)** Genus, **(F)** Species.

Based on the species annotation data obtained from groups A and B, we further analyzed the Taxon Tags associated with the existing species annotations. It can be seen that as the species level range continues to shrink, the number of TAGs annotated to this level also decreases, and the overall proportion of the two groups has little change, but the overall number group B is larger than group A. However, the overall proportion does not change much. However, when the level becomes more refined to “species,” the proportion changes significantly. The number of tags with clear annotations is less than half, and even in group B, it only accounts for 6% (Figure 3). Meanwhile, when we further analyzed the gut microbiota of loaches under different feeding patterns, we found that: At the Phylum level (Figure 4A), Proteobacteria (A: 84.25%; B: 70.94%) and Bacteroidota (A: 7.87%; B: 24.32%) was the dominant intestinal microflora in both the control and fermentation groups. At the Class level (Figure 4B), Gammaproteobacteria (A: 84.12%; B: 70.89%) and Bacteroidia (A: 7.87%; B: 24.32%) were significantly distinguishable between the two groups and were also the top two dominant bacteria in both groups. At the Order level (Figure 4C), the top five remarkable bacterial orders in Group A were: Pseudomonadales (42.20%), Alteromonadales (35.58%), Flavobacteriales (4.42%), Bacteroidales (3.40%), and Aeromonadales (3.50%). However, the top five dominant bacterial orders in Group B were: Pseudomonadales (63.24%), Flavobacteriales (20.82%), Bacteroidales (3.45%), Alteromonadales (3.23%) and Lactobacillales (2.05%). At the Family level (Figure 4D), the dominant families in Group A were Shewanellaceae (35.58%), followed by Pseudomonadaceae (21.70%) and Moraxellaceae (20.38%), while the dominant families in Group B were Moraxellaceae (40.18%), Pseudomonadaceae (23.04%) and Flavobacteriaceae (20.69%). At the Genus level (Figure 4E), the dominant genera in Group A were Shewanella (35.58%), Pseudomonas (21.61%), and Acinetobacter (20.39%). The dominant genera in Group B were Acinetobacter (40.18%), Pseudomonas (22.48%), and Myroides (20.57%). At the Species level (Figure 4F), Shewanella (35.06%) and Akkermansia (2.93%) were tremendously enriched in Group A, and Shewanella (3.19%) was enormously enriched in Group B. At the same time, in order to more vividly demonstrate the expression and distribution of this species among different groups, we used clustering heatmaps and circos to further analyze the inter group relationships from “phylum” to “species” (Figures 5, 6). Although the dominant bacteria in Group A and Group B were the same in phylum and class, their abundance was different. As the classification became more and more detailed, the proportion and species difference between Group A and Group B were increasingly significant. Based on the above analysis of the intestinal flora of the loach, what can be apprehended is that the soybean fermentation liquid has a noticeable effect on the intestinal flora of the loach.

### 3.3 The diversity of intestinal microflora in loach

To further explore the differences in the richness and evenness of intestinal flora between Group A and Group B, we carried out α diversity analysis. A-diversity analysis refers to the abundance and diversity of species in a particular habitat or ecosystem, which can indicate the degree of isolation of species in the habitat, two important indexes, species richness (species status) and species evenness (distribution status), are customarily used to calculate.

The Chao1 index was used to measure the number of OTU in the reactive community, and the index was adapted to measure the number of OTU in the reactive community. The index of Group B was lower than that of Group A. Shannon index not only reflects community abundance but also diversity. The higher the Shannon index is, the higher diversity is. The Shannon index of Group A was 3.8137, and the Shannon Index of Group B was 3.5020, which indicated that the diversity of Group A was higher than that of Group B. The goods coverage scores of the sequencing data for both groups were more outstanding than 99.8%, indicating that the sequencing depth of these two groups of samples was sufficient to provide a more reliable description of the relationship of the microbiota between the different groups of samples (Table 2).

**TABLE 2:** Diversity index analysis.

Index	Shannon	Chao1	ACE	Goods coverage	Pielou	PD
A	3.813720	939.414	967.624	0.998654	0.387234	116.31911
B	3.501991	912.678	935.887	0.998823	0.357019	112.60938

Shannon, Chao1, ACE, Goods coverage, Pielou, and PD, which is mainstream alpha diversity index and their correlation analysis results.

**FIGURE 7:**
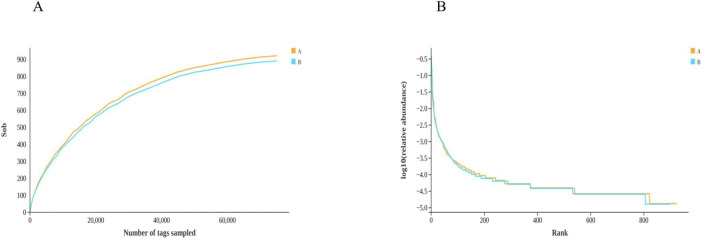
Alpha-diversity index analysis. **(A)** Rarefaction curve, **(B)** RankAbundance. Groups A and B in the figure represent the control and soybean fermented liquid, respectively.

Based on the Rarefaction curve (Figure 7A), the two sets of curves tend to flatten, indicating that the sequencing data are reasonable and the sequencing depth is reliable. The sample size and the library capacity provided in this experiment are large enough to represent the majority of bacteria in the gut flora of loach, and the species richness and library diversity reach saturation. The sequencing results can be acquainted with analyzing the discrepancy of intestinal flora. The Rarefaction curve showed that group A had a higher abundance of intestinal flora. The Rank Abundance curve (Figure 7B) can be used to measure the richness and evenness of the categories contained in the sample. That is, in the horizontal direction, the abundance of the category is reflected by the width of the curve. The higher the abundance of the category, the larger the span of the curve on the horizontal axis. In the figure, the span of the Group A curve on the horizontal axis is weightier than that of Group B, indicating that the abundances of Group A are higher, the more homogeneous the distribution of species, the smoother the curves of Group A and Group B are similar, which shows that the uniformity of classification of the two groups is correspondent. The Rarefaction curve and Rank Abundance curve showed that Group A had a higher Abundance of intestinal flora. We speculate that the reduction of intestinal microbiota in group B might be for the repair of microflora dysregulation. While Group B had a lower gut microbiota abundance, Group B had an increase in beneficial bacteria such as Flavobacteriaceae, suggesting that soy fermentation could improve the gut microbiota of loach.

### 3.4 Prediction of intestinal microflora in loach

**FIGURE 8:**
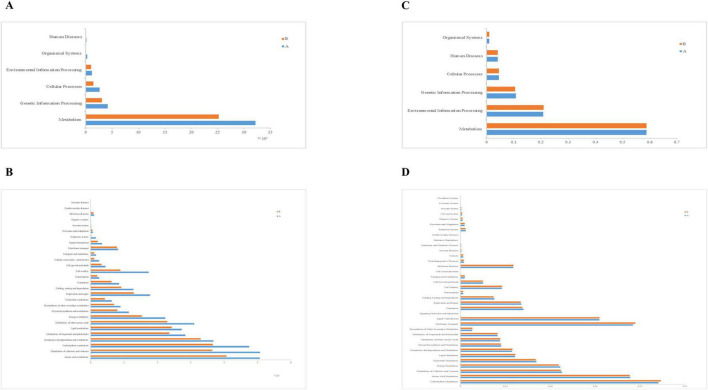
Function annotation, relative abundance of predicted function for specific KEGG modules (level 1 and level 2). **(A)** Relative abundance map for predicting PICRUSt function in the 1st level midgut, **(B)** Relative abundance map for predicting PICRUSt function in the 2st level midgut, **(C)** Relative abundance map of Tax4Fun function prediction in level 1 midgut, **(D)** Relative abundance map of Tax4Fun function prediction in level 2 midgut.

**FIGURE 9:**
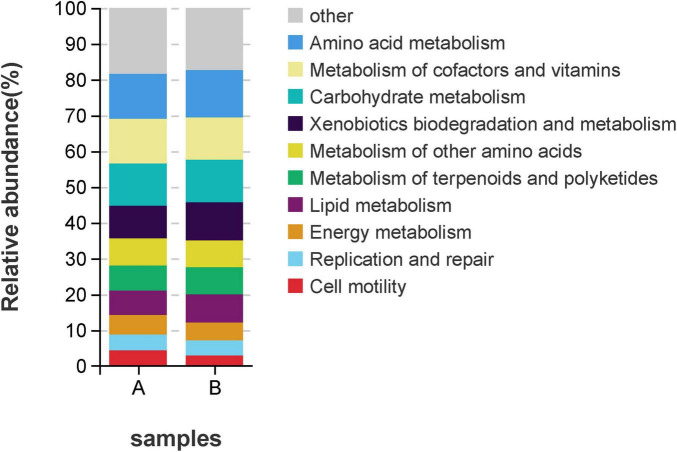
PICRUSt Level 2 Function annotation, x-axis represents group, y-axis represents relative abundance.

To explore the functional information of intestinal flora in two groups of loach, we used PICRUSt and Tax4Fun functions to predict. Firstly, in the first level structure of PICRUSt, we found that the obtained OTUs were annotated to six levels, namely “Metabolism,” “Genetic Information Processing,” “Cellular Processes,” “Environmental Information Processing,” “Organismal Systems.” Among them, we found that metabolic OTUs accounted for the majority, followed by genetic information processing, and finally “Human Diseases.” In both groups, the OTUs annotated in Group A were consistently higher than those in Group B (Figure 8A). Meanwhile, the relative abundance of metabolism significantly decreased in Group B with the addition of soybean fermentation broth, by 21.6%. In the PICRUST community function prediction at level 2 (Figures 8B, 9), we found that the top 10 significantly different metabolic pathways were “Amino acid Metabolism,” “Metabolism of cofactors and vitamins,” “Carbohydrate Metabolism,” “Xenobiotics biodegradation and Metabolism,” “Metabolism of other Amino acids,” “Metabolism of terpenoids and polyketides,” “Lipid Metabolism,” “Energy Metabolism,” “Replication and repair” and “Cell motility.” In level 2 (Figure 8B), group A and group B were involved in “amino acid metabolism,” “Carbohydrate metabolism,” “Metabolism of terpenoids and polyketides.” There is a significant difference in the above, and all three pathways belong to the Metabolism level. In addition, based on Figure 9, we found that compared to Group A, Group B has fewer annotations for cell movement pathways and more annotations for heterologous biodegradation and metabolic pathways.

**FIGURE 10:**
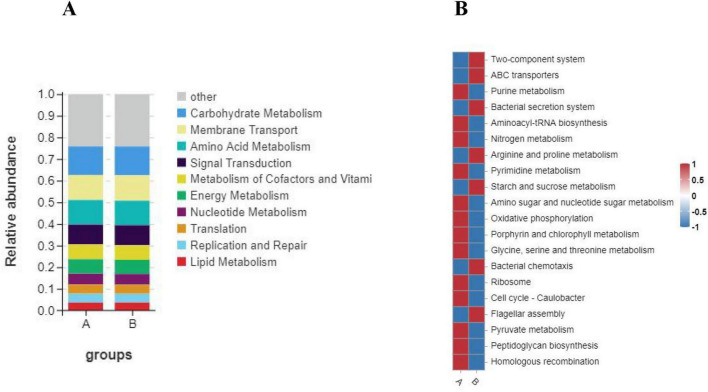
Functional annotation clustering heat map using Tax4Fun on level 2 **(A)** and level 3 **(B)**. A: The color represents the type of function, and the vertical axis represents the percentage of each function in the group. B: The color scale reveals indicates the level of abundance in two groups. Groups A and B in the figure represent the control and soybean fermented liquid, respectively.

Then we annotated the functions of KEGG Pathway using Tax4Fun software and calculated the abundance information of each Pathway and KO ID. We also obtained six levels of information, namely: “Metabolism,” “Environmental Information Processing,” “Genetic Information Processing,” “Cellular Processes,” “Human Diseases,” “Organismal Systems.” Through statistics, we found that there were only slight differences between group A and group B in Tax4fun prediction, and group A was slightly higher than group B only in the Metabolism and Genetic Information Processing pathways (Figure 8C). Then, in the tax4Fun secondary prediction, we identified 36 annotation pathways (Figure 8D). By analyzing the top 10 significantly different pathways (Figure 10A), that is: “Carbohydrate Metabolism,” “Membrane Transport,” “Amino Acid Metabolism,” “Signal Transduction,” “Metabolism of Cofactors and Vitamins,” “Energy Metabolism,” “Nucleotide Metabolism,” “Translation,” “Replication and Repair” and “Lipid Metabolism,” the abundance of intestinal microflora in Group B was slightly lower than that in Group A. The results showed that there stand six distinct ways to predict the intestinal microflora of Group A and Group B loach by using these two methods, “Metabolism of cofactors and vitamins,” “Carbohydrate Metabolism,” “Lipid Metabolism,” “Energy Metabolism,” “Replication and repair” and “Amino acid Metabolism.” At level 3 (Figure 10B), 20 pathways were significantly enriched. As for Group B, its relative abundance increased extraordinarily in seven significant enrichment pathways, namely, “Two-component system,” “ABC transporters,” “Bacterial secret system,” “Arginine and profile metabolism,” “Starch and sucrose metabolism,” “Bacterial chemotaxis” and “Flagellar assembly.” And for Group A, its relative abundance significantly increased in 13 enriched pathways, such as “Purine metabolism,” “Aminoacyl-tRNA biosynthatisis,” “Nitrogen metabolism,” “Pyrimidine metabolism” and “Amino sugar and nucleoside sugar metabolism.”

### 3.5 Phenotypic contribution analysis

**FIGURE 11:**
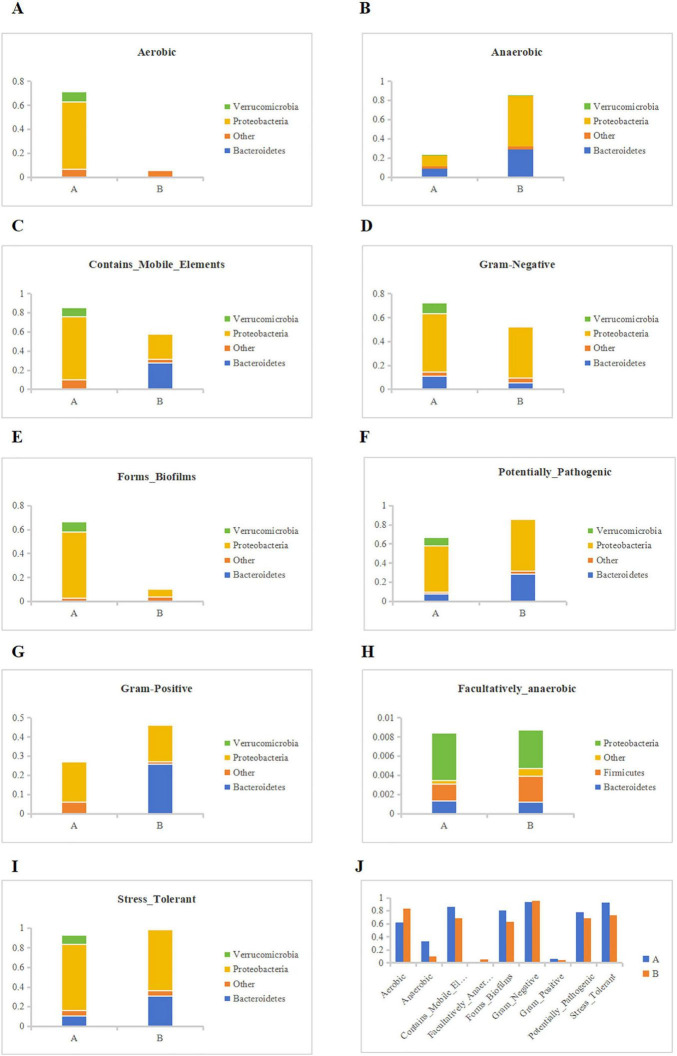
Bugbase phenotype classification prediction. **(A)** Contribution of each group to Aerobic, **(B)** Contribution of each group to Anaerobic, **(C)** Contribution of each group to Contains_Mobile_Elements, **(D)** Contribution of each group to Gram-Negative, **(E)** Contribution of each group to Forms_Biofilms, **(F)** Contribution of each group to Potentially_Pathogenic, **(G)** Contribution of each group to Gram Positive, **(H)** Contribution of each group to Facultatively_anaerobic, **(I)** Contribution of each group to Stress_Tolerant, **(J)** Phenotypic abundance results predicted by BugBase.

We conducted Bugbase phenotype classification prediction based on the Greengenes species annotation abundance table. Starting from different types of communities, explore the abundance of type A and B in different microbial communities. Among the three types of Oxygen Maximizing, we found that the proportion of aerobic bacteria in group A was significantly higher than that in group B, while the opposite was true in the anaerobic type. At the same time, the proportion of facultative anaerobic bacteria in the two groups was not significantly different, and verrucomicobia was not found in group B (Figure 11H). Through a detailed investigation of Aerobic, we found that neither group A nor group B had proteobacteria. In addition, compared to group A, group B targeted a relatively single and low proportion of bacteria (Figure 11A). In the anaerobic group, we found the opposite situation, with group B having a significantly higher abundance of bacteria than group A (Figure 11B). Meanwhile, in Academically anaerobic, the abundance of groups A and B is similar, but group B is slightly higher than group A. Later, in Mobile Element Containing, we found that the abundance of group B decreased compared to group A, and also found that group B may affect bacterioidetes (Figure 11C). For Biofilm Forming, we found that the number of proteobacteria significantly decreased in group B with soybean fermentation broth added (Figure 11E); For Pathogenic, we found that Group B may have a greater impact on Pathogenic, and compared to Group A, it is more likely to have an effect on Bacteroidetes (Figure 11F); For Oxidative Stress Tolerance, we found that there was not much difference in abundance between the two groups (Figure 11I); Finally, for Gram positive and Gram negative bacteria, Group B contributed more to them than Group A (Figures 11D, G). In summary, we speculate that the use of soybean fermentation broth may exert its effects by affecting verrucomicrobia and bacteroidetes. Finally, we summarized the phenotypic abundance of 9 types and found that soybean fermentation broth can exert anti-inflammatory effects by reducing pathogenic bacteria, anaerobic bacteria, oxidative stress tolerant bacteria, etc., (Figure 11J).

## 4 Discussion

Animal intestinal tract is not only an important place for digestion and absorption, but also one of the largest immune organs, which plays an extremely important role in the maintenance of normal immune defense function (Li C. et al., 2025). As the largest and most complex microecological environment of the human body, the intestinal microbes themselves also play a role in maintaining health and repairing damage. As a aquaculture value of aquatic loach, one of the most common factors affecting its growth performance is enteritis. Studies have shown that the gut microbiota structure is significantly different between enteritis animals and normal animals, with obvious intestinal microbiota disorders (Guo et al., 2025).

Fermentation broth has been shown to affect the composition of the intestinal microflora (Adebo and Gabriela Medina-Meza, 2020; Gumienna et al., 2025; Li Q. et al., 2025; Littman and Pamer, 2011; Zhang et al., 2025). However, research data related to aquatic animals are lacking. In this study, we performed high-throughput sequencing of the loach midgut by using LPS to induce inflammatory responses, and found that the diversity and richness of LPS infected loach changed in treated and untreated groups. Previous studies have found that disease status (inflammatory bowel disease, metabolic syndrome) may change the composition of flora, resulting to excessive abundance of flora (Littman and Pamer, 2011). Our study shows that after the treatment of soybean fermentation liquid, the intestinal flora richness of loach can be reduced to a certain extent, so the imbalance of loach intestinal flora induced by LPS can be repaired to a certain extent, reduce pathogenic bacteria and increase beneficial bacteria. Proteobacteria are frequently found to increase in disease and have been identified by some authors as a possible marker of microbiota instability and, thus, predisposing to disease onset (Maharshak et al., 2013; Selvanantham et al., 2016; Shin et al., 2015). At the same time, gut microbiota dysbiosis can significantly increase the levels of proinflammatory cytokines IL-6 and F/B ratio, induce inflammation, and the increased proportion of Firmicutes/Bacteroidota (F/B) is considered to be a marker of gut microbiota disorder (Evans et al., 2014). We studied the composition of the gut microbiota in the two groups and found that the taxonomic levels varied among the phyla, classes, order, family, genus, and species. At the gate level, the first three dominant bacterial phyla were (A: 84.25%; B:70.94%), Bacteroidota (A:7.87%; B:24.32%), Firmicutes (A: 3.37%; B: 2.98%), which are consistent with the results of other studies (Pilla and Suchodolski, 2019; Wang W. et al., 2024). It can be seen that the relative abundance of Proteobacteria, which is prone to disease, has a significant downward trend, However, the proportion of Bacteroidota was significantly higher in the treatment group. We believe that the increased proportion of beneficial bacteria in the Bacteroidota phylum causes, Also we found that after the use of soy fermentation broth, The proportion of Firmicutes/Bacteroidota (F/B) has decreased significantly, Therefore, it is believed that soybean fermentation liquid can improve the intestinal flora disease induced by LPS and inhibit inflammation. At the family level, we observed a significant reduction in the relative abundance of pathogenic bacteria, such as Shewanella and Aeromonadaceae, alongside a notable increase in beneficial bacteria, including Lactobacillus and Muribaculaceae. Research has demonstrated that Lactobacillus, a genus known for its probiotic properties, plays a crucial role in maintaining intestinal health by fermenting carbohydrates to produce short-chain fatty acids (SCFAs), enhancing the host immune response, and combating harmful microorganisms. Similarly, Muribaculaceae, a family within the order Bacteroidales, has been shown to support gut health by suppressing the expression of pro-inflammatory cytokines, thereby promoting a balanced intestinal environment (Dong et al., 2025; Ibrahim et al., 2025; Pereira et al., 2020; Qi et al., 2017; Zhao et al., 2025). Through PICRUSt and Tax4Fun function prediction, we found that bacterial community functions were related to metabolic pathways, mainly enriched in “Metabolism of Cofactors and Vitamins,” “Carbohydrate Metabolism,” “Lipid Metabolism,” “Xenobiotics Biodegradation and Metabolism,” “Metabolism of Other Amino Acids,” “Metabolism of Terpenoids and Polyketides,” and “Biosynthesis of Other Secondary Metabolites.” This indicates that soybean fermentation liquid can play a role in maintaining the balance of intestinal flora by affecting the nutrient metabolism level of loach.

## 5 Conclusion

In conclusion, this study showed that soybean fermentation broth is able to reshape the intestinal microbiota of loach by increasing the number of beneficial bacteria such as Muribaculaceae, Lactobacillus, reducing the number of pathogenic bacteria such as Shewanella, Aeromonas, and repairing the inflammatory response induced by LPS. At the same time, through function prediction, we found that soybean fermentation broth can play a role in maintaining the balance of intestinal flora by affecting the nutritional metabolism level of loach, which will provide new insights for the future study of diseases caused by intestinal bacterial disorders, and will also help us to further promote the process of studying the pathophysiology of intestinal inflammation. But the mechanisms of these changes still need to be further explored.

## Data Availability

The data presented in the study are deposited in the genebank repository, accession number PRJNA1230112.
